# The Association of Ageist Attitudes With All-Cause Hospitalizations and Mortality

**DOI:** 10.1177/2333721419892687

**Published:** 2019-12-03

**Authors:** Yaqub Nadeem Mohammed, Juliana Ferri-Guerra, Douglas Salguero, Dhanya Baskaran, Raquel Aparicio-Ugarriza, Michael J. Mintzer, Jorge G. Ruiz

**Affiliations:** 1 Miami VA Healthcare System Geriatric Research, Education and Clinical Center (GRECC), FL, USA; 2 Miller School of Medicine, University of Miami, FL, USA; 3 Florida International University, Miami, Florida, USA

**Keywords:** ageism, explicit ageism, implicit ageism, hospitalizations, mortality

## Abstract

**Background:** Ageism is the systematic stereotyping and discrimination against older adults. Explicit ageism involves conscious control and implicit ageism involves unconscious processes. Studies have shown that ageist attitudes may be associated with poor clinical outcomes like hospitalizations and mortality. **Objective:** Determine the association of explicit and implicit ageism with all-cause hospitalizations and mortality in a sample of Veterans. **Method:** Retrospective cohort study of community-dwelling Veterans 50 years and older who underwent evaluations of explicit ageism using Kogan’s Attitudes Toward Old People Scale and implicit ageism assessed with Implicit Association Test (IAT) during July 2014 to April 2015 and were followed until 2018. Data on all-cause hospitalizations and mortality following the initial assessment of ageism was aggregated. **Results:** The study included 381 participants, 89.8% male, 48.0% White, and mean age was 60.5 (*SD* = 7.2) years. A total of 339 completed the IAT. Over a mean follow-up of 3.2 years (*SD* = 0.3), 581 hospitalizations, and 35 deaths occurred. Neither explicit nor implicit ageism was associated with an increased risk for all-cause hospitalization or mortality on follow-up. **Discussion:** Future research may benefit from investigating whether ageist attitudes may predict all-cause hospitalizations and mortality in longitudinal studies including more diverse samples.

## Background

Ageism is the systematic stereotyping of and discrimination against older adults (Butler, 1969). Unlike other forms of prejudice, ageism is considered more socially acceptable (Palmore, 2003) and seems to be the strongest among all the studied social biases (Nosek et al., 2002). Longitudinal studies demonstrate that having negative stereotypes and attitudes toward older adults at younger ages were associated years later with cardiovascular disease (Levy et al., 2009), memory impairment (Levy et al., 2012), decreased capacity to recover from disability (Levy et al., 2012), hearing loss (Levy et al., 2006), diminished will to live (Levy et al., 1999), lower participation in preventive activities (Levy & Myers, 2004), lower perception of functional health (Levy et al., 2002), and poor recovery after myocardial infarction (Levy et al., 2006). Furthermore, these problems individually or in combination may have increased the risk for all-cause hospitalization by 50% (Levy et al., 2015), and reduced longevity (Levy et al., 2002) compared with people who harbored more positive explicit attitudes toward old age. There is no comparable longitudinal data on individuals with implicit ageist attitudes. Although many studies have focused on the ageist attitudes of health care professionals and their pernicious effects on older patients, this and other longitudinal studies focus on individuals’ attitudes toward their own aging process. Two theories may serve to explain these findings. The theory of stereotype embodiment proposes that a lifetime exposure to widespread ageism leads to older persons’ internalization of ageist messages which then become part of their unconscious beliefs about older persons in general (Levy, 2009). The stereotype threat theory posits that under specific conditions involving widely held stereotypes, older persons would act subconsciously to fulfill those stereotypes, even when the outcome becomes detrimental to themselves (Lamont et al., 2015).

Devine (1989) studied prejudice using dual process models that include explicit and implicit levels. Explicit bias involves deliberative, controlled processes. In contrast, implicit bias involves automatic, unconscious processes. Explicit biases are assessed through surveys and questionnaires whereas implicit biases are assessed with measures of automatic behavior such in the Implicit Association Test (IAT; Greenwald et al., 1998). However, evidence shows that explicit, self-report measures may be influenced by social expectations, political correctness, answering style, interpretations of individual item wording, or limits of participant memory (Amodio & Devine, 2006; Dovidio et al., 2002; Puhl & Brownell, 2006; Puhl et al., 2005; Schwartz et al., 2003; Vanman et al., 1997). Most studies dealing with the longitudinal outcomes of ageism have used explicit measures. Implicit bias, however, may better predict behaviors toward older people than self-report measures (Greenwald et al., 2009; Roddy et al., 2011). The IAT is the most well-known and used implicit measure (Greenwald et al., 1998). The IAT evaluates the overall strength of associations between concepts by ascertaining individuals’ reaction time (latency) in response to categorization tasks (Greenwald et al., 2002). In many instances, both controlled and automatic processes are operating independently and concurrently (Greenwald et al., 2003) yet may display opposite bias. Explicit and implicit attitudes may contribute to higher risk for poor clinical outcomes and higher health care utilization.

The purpose of the current study was to determine the association of explicit and implicit ageism with all-cause hospitalizations and mortality in a sample of Veterans. This study predicted that explicit and implicit ageist attitudes will be associated with a greater risk for all-cause hospitalizations and mortality after adjustment for covariates.

## Method

### Design and Participants

This is a retrospective cohort study of community-dwelling Veterans 50 years and older who were receiving outpatient care at a VA (U.S. Department of Veterans Affairs) facility between July 2014 and April 2015. Veterans were included who met the following criteria: enrollment in a VA clinic, cognitively intact (Mini-Cog of > 3), and nondepressed (Patient Health Questionnaire–2 [PHQ-2] of < 3), and were followed for up to 3 years. After obtaining an exempted review status from the institutional review board (IRB) department in 2013, an addendum was submitted in 2018 to conduct a retrospective electronic health record review to obtain data from these participants to determine all-cause hospitalizations and mortality. All patients signed an informed consent and the study was done following the Declaration of Helsinki.

### Measures

All consented eligible participants completed online versions of a sociodemographic questionnaire and the following instruments.

#### Kogan’s Attitudes Toward Old People (KAOP) Scale

The scale measures explicit attitudes toward older persons. It is easy to score and fast to complete. It assesses participants’ opinions and stereotypes regarding older adults: their intellectual abilities, image, levels of dependence, personality, living situation, personal appearance, influence, and the individuals’ feelings of discomfort in the presence of older adults. The instrument consists of 17 matched pairs of positive and negative statements about older individuals. Responses are rated on a 6-point Likert-type scale that ranges from *strongly agree* to *strongly disagree*. The possible scores range between 34 and 204 with higher scores representing more positive attitudes toward older individuals (Kogan, 1961). A score of 102 indicates a neutral attitude (Kearney et al., 2000).

#### The IAT

**Figure 1.**
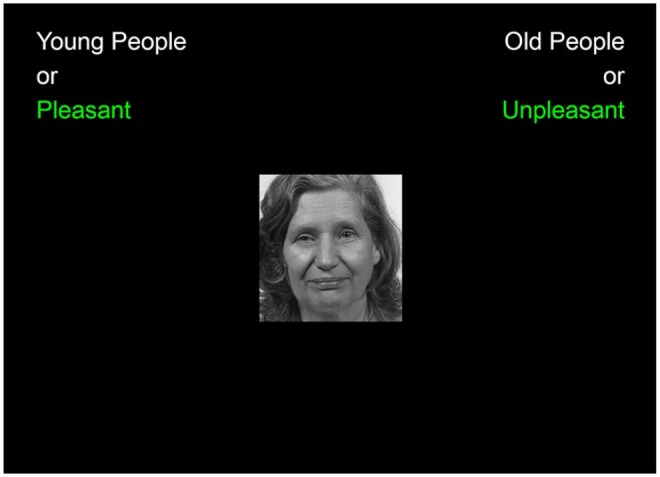
Implicit Association Test screenshot. *Source.* Information Technology Laboratory by FERET (2019), https://www.nist.gov/itl/products-and-services/color-feret-database. Adapted from the Implicit Association Test using Inquisit by Millisecond from https://www.millisecond.com/products/inquisit5/laboverview.aspx.

To measure implicit bias, participants completed an online version of the IAT (Inquisit Millisecond Software, Seattle, WA). Participants are asked to pair the terms “Old People” and “Young People” with both positive (a total of 10 words) and negative (a total of 10 words) “affective” attributes, such as unpleasant–pleasant. The words are combined with 10 photographs of older individuals and 10 photographs of younger individuals according to established IAT protocols (Figure 1). Next, each participant completed an evaluative IAT (Greenwald et al., 1998), in which they paired pleasant and unpleasant words with pictures of old and young persons (Table 1). Target category and attribute labels remained on the top left and top right of the screen throughout the task, while stimulus pictures and words appeared at the center of the screen. A red “X” appeared whenever participants made an error, which they were required to correct before moving onto the next trial. Latencies were recorded to the correct response. Participants were instructed to make their classifications as quickly and accurately as possible. Greenwald developed an algorithm for scoring of the IAT, which is a timed word classification task (Greenwald et al., 2003).

**Table 1.** Implicit Association Test.

Response keys				
Block	No. of trials (Practice/Critical)	Task	Left key^a^	Right key^b^
1	10 (Practice)	Word discrimination	Pleasant	Unpleasant
2	10 (Practice)	Face discrimination	Young	Old
3	20 (Critical)	Combined word and face discrimination	Pleasant/young	Unpleasant/old
4	20 (Critical)	Combined word and face discrimination	Pleasant/young	Unpleasant/old
5	10 (Practice)	New keys (Switched)	Old	Young
6	20 (Critical)	Combined word and face discrimination	Pleasant/old	Unpleasant/young
7	20 (Critical)	Combined word and face discrimination	Pleasant/old	Unpleasant/young

*Source.* Adapted from Inquisit Millisecond Software, https://www.millisecond.com/download/library/v5/iat/ageiat/ageiat.web.

a Left Key = “E.” ^b^ Right Key = “I.”

In the present study, positive IAT *d* scores indicated stronger associations of negative attributes with old people compared with young people, whereas an IAT *d* score of 0 indicated no difference in associations with old people compared with young people. IAT *d* scores were categorized into preference for older individuals (≤.15), neutral (>−.15, ≤.15), slight (IAT *d* score > .15), moderate (IAT *d* score > .35), or strong (IAT *d* score ≥ .65) preference for younger individuals (Greenwald et al., 2003).

#### Outcome variables

The two primary study outcomes were hospitalization and mortality.

#### Hospitalizations

Following the initial assessment of explicit and implicit ageism, patients were followed through September 30, 2018. Data on all-cause hospitalizations at the VA was obtained from the VA Corporate Data Warehouse (CDW). The number of hospital admissions for medical, mental health, and surgical teams were captured during the previous year and prospectively for the follow-up period. Trained research staff recorded the primary diagnosis for hospitalization by International Classification of Diseases codes from inpatient treatment files; the primary reason for hospitalization was grouped by diagnosis.

#### Mortality

All-cause mortality was verified via official source including VHA (Veterans Health Administration) facilities, death certificate, and National Cemetery Administration available from the VA CDW. There is high agreement (91%–99%) between dates of death recorded in the CDW and dates of death recorded in external sources that feed the VHA Vital Status File. The last day of follow-up was September 30, 2018.

### Data Analysis

Baseline characteristics are presented as frequency (percent) for categorical variables, as *M* ± *SD* for normally distributed continuous variables, and as median (interquartile range [IQR]) for continuous variables with skewed distributions. We report descriptive statistics of age, education, marital status, race, ethnicity, median household income, number of medications, body mass index (BMI), and Charlson comorbidity index (Charlson et al., 1987). The 5-Digit ZIP Code Tabulation Area (ZCTA) was used to determine the median household income in the past 12 months (in 2011 inflation-adjusted dollars) by racial groups (U.S. Census Bureau, 2007–2011). We compared the mean scores of KAOP and IAT using one-way analysis of variance (ANOVA) and comparisons of proportions were carried out using the Pearson chi-square test of homogeneity. The association of explicit and implicit ageism with hospitalizations was determined with the Andersen–Gill model (Amorim & Cai, 2015), accounting for repeated hospitalizations performing univariate analysis, as well as multivariate analysis adjusting for age, race (White vs. Non-White), ethnicity (Hispanic vs. Non-Hispanic), median household income, age-adjusted Charlson comorbidity index, and hospitalizations in the previous year. The proportional hazard assumption was tested using scaled Schoenfeld residuals and was found to be valid. Follow-up duration in years was calculated as follows: (September 30, 2018 − ageism assessment administration date) / 365. Cox proportional hazards regression analysis was performed to calculate the hazard ratios (HRs) and 95% confidence intervals (CIs) of survival for scores of KAOP Scale and the IAT. Known factors associated with mortality (age, race, ethnicity, mean household income, and multimorbidity) were considered for inclusion in the adjusted analysis. Collinearity among covariates was quantified using the variance inflation factor, taking a cutoff of two as exclusion criterion. We did not exclude variable having a high collinearity among themselves. A Pearson correlation was run to assess the relationship between the KAOP Scale and IAT. Associations were considered significant if *p* < .05. All analyses were performed using the SPSS 24.0 for Macintosh (SPSS, Inc., Chicago, Illinois) and SAS version 3.71 (SAS Institute Inc., Cary, North Carolina). All statistical tests were two-tailed, and statistical significance was assumed for *p* < .05.

## Results

### Patient Characteristics

A total of 381 participants were included in the study. Patients were 89.8% male, 48.0% White, 87.9% non-Hispanic, and the mean age was 60.5 (*SD*= 7.2) years. Table 2 shows participant characteristics. The KAOP scores revealed that 364 (95.5%) of the 381 participants showed a general positive attitude toward older people (scores > 102). In contrast, of the 339 participants completing the IAT, scores showed that 22 (5.8%) preferred older people, 32 (8.4%) were neutral, 43 (11.3%) had a slight, 63 (16.5%) had a moderate, and 179 (47.0%) had a strong preference for younger individuals. There was no significant correlation between the KAOP and the IAT scores (*r* = .043, *p* = .431).

**Table 2.** Participant Characteristics.

Variables	Total (*n* = 381, 100%)
Age, *M* (*SD*)	60.51 (7.16)
Male, *n* (%)	342 (89.76)
Caucasian, *n* (%)	183 (48.03)
Not Hispanic^a^, *n* (%)	335 (87.93)
Married, *n* (%)	91 (23.90)
Mean household income, *M* US$ (*SD*)	45,942 (18,375)
More than five medications, *n* (%)	195 (51.18)
Charlson Comorbidity Index, *M* (*SD*)	3.80 (1.87)
Kogan Scores, *M* (*SD*)	121.13 (11.90)
IAT scores^a^, *M* (*SD*)	0.6208 (0.4746)

*Note. n* = number of participants; IAT = Implicit Association Test.

a Data available for 339 participants.

### Hospitalizations

There were 584 all-cause hospitalizations over a median follow-up period of 1,440 days (IQR = 100) with the range between 0 and 47 hospital admissions. Over the follow-up period, 200 participants (52.5%) did not have any hospitalizations whereas 181 (47.5%) had at least one hospitalization. As shown in Table 3, using the Andersen–Gill model for recurrent-event analysis of hospitalizations, fully adjusted for covariates, including age, race, ethnicity, median household income, Charlson Comorbidity Index, and hospitalizations in the previous year, neither explicit ageism as measured with the KAOP, adjusted HR = 1.01 (95% CI = [0.99, 1.03]),*p* = .286, nor implicit ageism as measured with the IAT scores were associated with risk for hospitalizations, adjusted HR = 1.05 (95% CI = [0.69, 1.59]), *p* = .824.

**Table 3.** Association Between Explicit (KAOP) and Implicit Ageism (IAT) and Risk of Hospitalizations at Follow-Up.

Measures	Unadjusted [95% CI]	*p* value	Adjusted hazard ratio [95% CI]	*p* value
Explicit ageism (Kogan)	1.01 [0.99, 1.03]	.339	1.01 [0.99, 1.03]	.286
Implicit ageism (IAT)^a^	0.98 [0.64, 1.49]	.928	1.05 [0.69, 1.59]	.824

*Note.* Adjusted for age, race, ethnicity, median household income, Charlson Comorbidity Index, and hospitalizations in the previous year. KAOP = Kogan’s Attitudes Toward Old People; IAT = Implicit Association Test; CI = confidence interval.

a Data available for 339 participants.

### Mortality

Over the median follow-up period 35 deaths occurred. As shown in Table 4, using a Cox’s regression analysis fully adjusted for covariates, including age, race, ethnicity, median household income, and Charlson Comorbidity Index, neither KAOP scores nor IAT scores were associated with risk of all-cause mortality during follow-up, HR = 1.02 (95% CI = [0.99, 1.05]), *p* = .157, and HR = 0.72 (95% CI = [0.34, 1.51]), *p* = .380, respectively (Table 4).

**Table 4.** Association Between Explicit (KAOP) and Implicit Ageism (IAT) and Risk of Mortality at Follow-Up.

Measures	Unadjusted [95% CI]	*p* value	Adjusted hazard ratio [95% CI]	*p* value
Explicit ageism (Kogan)	1.02 [0.99, 1.04]	.180	1.02 [0.99, 1.05]	.157
Implicit ageism (IAT)^a^	0.69 [0.34, 1.40]	.301	0.72 [0.34, 1.51]	.380

*Note.* Adjusted for age, race, ethnicity, median household income, and Charlson Comorbidity Index. KAOP = Kogan’s Attitudes Toward Old People; IAT = Implicit Association Test; CI = confidence interval.

a Data available for 339 participants.

## Discussion

The predicted hypothesis that explicit ageism measured with the KAOP Scale and implicit ageism as measured with the IAT would be associated with a greater risk for increased rate of all-cause hospitalizations and mortality was rejected. Neither explicit nor implicit ageism were associated with an increased risk for all-cause hospitalization or mortality on follow-up. The KAOP and IAT scores were not correlated: The overwhelmingly majority of participants showed a favorable explicit attitude while on the contrary more than three quarters showed negative implicit bias toward older individuals.

Only one longitudinal study with 10-year follow-up showed an association of explicit ageist attitudes with a 50% higher risk of hospital admission in those individuals with negative attitudes to aging (Levy et al., 2015). A total of 231 participants answered a single question from the Images of aging instrument (explicit measure) and were followed over time for the occurrence of hospitalizations verified by self-report. In contrast, this study included a larger sample size of a predominantly male sample of Veterans who completed evaluations of explicit and implicit ageist attitudes. Although also longitudinal, this study had a much shorter follow-up period. Another stronger factor is that the average age of this sample was 16 years younger than that of Levy’s study, a factor that may account for the lower hospitalization and mortality rates during the shorter follow-up period. Another explanation relates to how explicit ageism was assessed in this study. Whereas we used the KAOP scale which measures individual attitudes toward older people in general, it may not be evaluating the domain of personal attitudes to oneself as the other scales do. That is, older persons may hold more positive views of themselves than the view they have of other, in their opinion, more “typical” older persons. This dissociation between attitudes toward one’s own aging as compared with other older persons has been reported (Celejewski & Dion, 1998; Rothermund & Brandtstädter, 2003). Measures that focus on self-perceptions of aging domains may be more likely associated with future clinical outcomes than measures like the KAOP.

There are no previous studies linking implicit ageism at baseline with subsequent hospitalizations and mortality. Levy et al. has shown in experimental studies that individuals’ exposure to subliminal implicit primes was associated with behavioral, psychological, and physiological changes (Hausdorff et al., 1999; Levy, 1996; Levy et al., 1999) but there was not follow-up or ascertainment of the effects on hospitalization or mortality. The findings of a lack of causality between IAT scores and subsequent all-cause mortality may suggest that implicit ageist attitudes may not result in the internalization of those ageist attitudes as purported by the stereotype embodiment and stereotype threat theories that will eventually contribute to poor clinical outcomes. Once again, that these effects will appear on follow-up is still a possibility, but the mean age of this cohort might have shown at least a trend. Another factor is that despite their high multimorbidity burden Veterans have access to an integrated health care system that provides a range of medical and social services (Nelson et al., 2014; Rosland et al., 2013) which may ameliorate the negative effects of ageist attitudes on their health care outcomes and utilization.

Strengths of this study include the large number of participants with complete assessments of explicit and implicit ageist attitudes, inclusion of complete health care data from electronic health records, and a relatively large sample with a reasonable follow-up period. There are certainly some limitations. This study used a convenience sample of male Veterans at one medical center data, and ethnic, racial, educational, and socioeconomic composition may be different from other Veterans’ facilities and other community-based health care institutions in the United States. Problems with the predictive validity of the IAT should cause us to be cautious in selecting this instrument and interpreting its results when evaluating implicit ageist attitudes. Education might influence ageist attitudes but was not considered as a covariate in the analyses. Future cohort studies should include more diverse, randomly selected samples from varied geographic locations and health care systems. Nevertheless, the results and conclusions can have important clinical implications and can motivate future clinical and epidemiological studies.

## Conclusion/Implications

The data suggest that neither explicit not implicit attitudes had an effect on all-cause hospitalizations or mortality in this sample of community-dwelling Veterans. Future research may benefit from longitudinal investigations of the impact of ageist attitudes in diverse populations on all-cause hospitalizations and mortality.

## References

[bibr1-2333721419892687] Amodio D. M. Devine P. G. (2006). Stereotyping and evaluation in implicit race bias: Evidence for independent constructs and unique effects on behavior. Journal of Personality and Social Psychology, 91(4), 652–661. 10.1037/0022-3514.91.4.652 17014291

[bibr2-2333721419892687] Amorim L. D. Cai J. (2015). Modelling recurrent events: A tutorial for analysis in epidemiology. International Journal of Epidemiology, 44(1), 324–333. 10.1093/ije/dyu222 25501468 PMC4339761

[bibr3-2333721419892687] Butler R. N. (1969). Age-ism: Another form of bigotry. The Gerontologist, 9(4), 243–246. 10.1093/geront/9.4_Part_1.243 5366225

[bibr4-2333721419892687] Celejewski I. Dion K. K. (1998). Self-perception and perception of age groups as a function of the perceiver’s category membership. International Journal of Aging & Human Development, 47(3), 205–216. 10.2190/GL4R-FJ7G-XGEK-MRR6 9879021

[bibr5-2333721419892687] Charlson M. E. Pompei P. Ales K. L. MacKenzie C. R. (1987). A new method of classifying prognostic comorbidity in longitudinal studies: Development and validation. Journal of Chronic Diseases, 40(5), 373–383. 10.1016/0021-9681(87)90171-8 3558716

[bibr6-2333721419892687] Devine P. G. (1989). Stereotypes and prejudice: Their automatic and controlled components. Journal of Personality and Social Psychology, 56(1), 5–18. 10.1037/0022-3514.56.1.5

[bibr7-2333721419892687] Dovidio J. F. Kawakami K. Gaertner S. L. (2002). Implicit and explicit prejudice and interracial interaction. Journal of Personality and Social Psychology, 82(1), 62–68. 10.1037/0022-3514.82.1.62 11811635

[bibr8-2333721419892687] Greenwald A. G. Banaji M. R. Rudman L. A. Farnham S. D. Nosek B. A. Mellott D. S. (2002). A unified theory of implicit attitudes, stereotypes, self-esteem, and self-concept. Psychological Review, 109(1), 3–25. 10.1037/0033-295X.109.1.3 11863040

[bibr9-2333721419892687] Greenwald A. G. McGhee D. E. Schwartz J. L. (1998). Measuring individual differences in implicit cognition: The Implicit Association Test. Journal of Personality and Social Psychology, 74(6), 1464–1480. 10.1037/0022-3514.74.6.1464 9654756

[bibr10-2333721419892687] Greenwald A. G. Nosek B. A. Banaji M. R. (2003). Understanding and using the Implicit Association Test: I. An improved scoring algorithm. Journal of Personality and Social Psychology, 85(2), 197–216. 10.1037/0022-3514.85.2.197 12916565

[bibr11-2333721419892687] Greenwald A. G. Oakes M. A. Hoffman H. G. (2003). Targets of discrimination: Effects of race on responses to weapons holders. Journal of Experimental Social Psychology, 39(4), 399–405. 10.1016/S0022-1031(03)00020-9

[bibr12-2333721419892687] Greenwald A. G. Poehlman T. A. Uhlmann E. L. Banaji M. R. (2009). Understanding and using the Implicit Association Test: III. Meta-analysis of predictive validity. Journal of Personality and Social Psychology, 97(1), 17–41. 10.1037/a0015575 19586237

[bibr13-2333721419892687] Hausdorff J. M. Levy B. R. Wei J. Y. (1999). The power of ageism on physical function of older persons: Reversibility of age-related gait changes. Journal of the American Geriatrics Society, 47(11), 1346–1349. 10.1111/j.1532-5415.1999.tb07437.x 10573445

[bibr14-2333721419892687] Kearney N. Miller M. Paul J. Smith K. (2000). Oncology healthcare professionals’ attitudes toward elderly people. Annals of Oncology, 11(5), 599–601. 10.1023/A:1008327129699 10907955

[bibr15-2333721419892687] Kogan N. (1961). Attitudes toward old people: The development of a scale and an examination of correlates. Journal of Abnormal and Social Psychology, 62(1), 44–54. 10.1037/h0048053 13757539

[bibr16-2333721419892687] Lamont R. A. Swift H. J. Abrams D. (2015). A review and meta-analysis of age-based stereotype threat: Negative stereotypes, not facts, do the damage. Psychological Aging, 30(1), 180–193. 10.1037/a0038586 PMC4360754 25621742

[bibr17-2333721419892687] Levy B. (1996). Improving memory in old age through implicit self-stereotyping. Journal of Personality and Social Psychology, 71(6), 1092–1107. 10.1037/0022-3514.71.6.1092 8979380

[bibr18-2333721419892687] Levy B. R. (2009). Stereotype embodiment: A psychosocial approach to aging. Current Directions in Psychological Sciences, 18(6), 332–336. 10.1111/j.1467-8721.2009.01662.x PMC2927354 20802838

[bibr19-2333721419892687] Levy B. R. Ashman O. Dror I. (1999). To be or not to be: The effects of aging stereotypes on the will to live. Omega—Journal of Death and Dying, 40(3), 409–420. 10.2190/Y2GE-BVYQ-NF0E-83VR 12557880

[bibr20-2333721419892687] Levy B. R. Myers L. M. (2004). Preventive health behaviors influenced by self-perceptions of aging. Preventive Medicine, 39(3), 625–629. 10.1016/j.ypmed.2004.02.029 15313104

[bibr21-2333721419892687] Levy B. R. Slade M. D. Chung P. H. Gill T. M. (2015). Resiliency over time of elders’ age stereotypes after encountering stressful events. Journal of Gerontology. Series B, Psychological Sciences and Social Sciences, 70(6), 886–890. 10.1093/geronb/gbu082 PMC4612340 24997287

[bibr22-2333721419892687] Levy B. R. Slade M. D. Gill T. M. (2006). Hearing decline predicted by elders’ stereotypes. Journal of Gerontology: Series B, Psychological Sciences and Social Sciences, 61(2), 82–87. 10.1093/geronb/61.2.p82 16497958

[bibr23-2333721419892687] Levy B. R. Slade M. D. Kasl S. V. (2002). Longitudinal benefit of positive self-perceptions of aging on functional health. Journal of Gerontology. Series B, Psychological Sciences and Social Sciences, 57(5), 409–417. 10.1093/geronb/57.5.P409 12198099

[bibr24-2333721419892687] Levy B. R. Slade M. D. Kunkel S. R. Kasl S. V. (2002). Longevity increased by positive self-perceptions of aging. Journal of Personality and Social Psychology, 83(2), 261–270. 10.1037//0022-3514.83.2.261 12150226

[bibr25-2333721419892687] Levy B. R. Slade M. D. May J. Caracciolo E. A. (2006). Physical recovery after acute myocardial infarction: Positive age self-stereotypes as a resource. The International Journal of Aging and Human Development, 62(4), 285–301. 10.2190/EJK1-1Q0D-LHGE-7A35 16739466

[bibr26-2333721419892687] Levy B. R. Slade M. D. Murphy T. E. Gill T. M. (2012). Association between positive age stereotypes and recovery from disability in older persons. Journal of the American Medical Association, 308(19), 1972–1973. 10.1001/jama.2012.14541 23168819 PMC3614078

[bibr27-2333721419892687] Levy B. R. Zonderman A. B. Slade M. D. Ferrucci L. (2009). Age stereotypes held earlier in life predict cardiovascular events in later life. Psychological Science, 20(3), 296–298. 10.1111/j.1467-9280.2009.02298.x 19222809 PMC2666386

[bibr28-2333721419892687] Levy B. R. Zonderman A. B. Slade M. D. Ferrucci L. (2012). Memory shaped by age stereotypes over time. Journal of Gerontology: Series B, Psychological Sciences and Social Sciences, 67(4), 432–436. 10.1093/geronb/gbr120 PMC3391075 22056832

[bibr29-2333721419892687] Nelson K. M. Helfrich C. Sun H. Hebert P. L. Liu C. F. Dolan E.. . . Fihn S. D. (2014). Implementation of the patient-centered medical home in the Veterans Health Administration: Associations with patient satisfaction, quality of care, staff burnout, and hospital and emergency department use. Journal of the American Medical Association, Internal Medicine, 174(8), 1350–1358. 10.1001/jamainternmed.2014.2488 25055197

[bibr30-2333721419892687] Nosek B. A. Banaji M. Greenwald A. G. (2002). Harvesting implicit group attitudes and beliefs from a demonstration web site. Group Dynamics: Theory, Research, and Practice, 6(1), 101–115. 10.1037//1089-2699.6.1.101

[bibr31-2333721419892687] Phillips P. J. Moon H. Rizvi S. A. Rauss P. J., (2000). The FERET Evaluation Methodology for Face-Recognition Algorithms. IEEE Trans. Pattern Anal. Mach. Intell., 22(10), 1090-1104. DOI: 10.1109/34.879790

[bibr32-2333721419892687] Palmore E. B. (2003). Ageism comes of age. The Gerontologist, 43(3), 418–420. 10.1093/geronb/gbv079

[bibr33-2333721419892687] Puhl R. M. Brownell K. D. (2006). Confronting and coping with weight stigma: An investigation of overweight and obese adults. Obesity, 14(10), 1802–1815. 10.1038/oby.2006.208 17062811

[bibr34-2333721419892687] Puhl R. M. Schwartz M. B. Brownell K. D. (2005). Impact of perceived consensus on stereotypes about obese people: A new approach for reducing bias. Health Psychology, 24(5), 517–525. 10.1037/0278-6133.24.5.517 16162046

[bibr35-2333721419892687] Roddy S. Stewart I. Barnes-Holmes D. (2011). Facial reactions reveal that slim is good but fat is not bad: Implicit and explicit measures of body-size bias. European Journal of Social Psychology, 41(6), 688–694. 10.1002/ejsp.839

[bibr36-2333721419892687] Rosland A. M. Nelson K. Sun H. Dolan E. D. Maynard C. Bryson C.. . . Schectman G. (2013). The patient-centered medical home in the Veterans Health Administration. The American Journal of Managed Care, 19(7), e263–e272. 23919446

[bibr37-2333721419892687] Rothermund K. Brandtstädter J. (2003). Age stereotypes and self-views in later life: Evaluating rival assumptions. International Journal of Behavioural Development, 27(6), 549–554. 10.1080/01650250344000208

[bibr38-2333721419892687] Schwartz M. B. Chambliss H. O. Brownell K. D. Blair S. N. Billington C. (2003). Weight bias among health professionals specializing in obesity. Obesity Research, 11(9), 1033–1039. 10.1038/oby.2003.142 12972672

[bibr39-2333721419892687] U.S. Census Bureau. (2007–2011). Income/earnings. https://www.census.gov/programs-surveys/acs/library/keywords/income.2017.html

[bibr40-2333721419892687] Vanman E. J. Paul B. Y. Ito T. A. Miller N. (1997). The modern face of prejudice and structural features that moderate the effect of cooperation on affect. Journal of Personality and Social Psychology, 73(5), 941–959. 10.1037/0022-3514.73.5.941 9364754

